# Manual pointing bias reflects spatial organization of number knowledge

**DOI:** 10.1038/s41598-026-39170-7

**Published:** 2026-02-11

**Authors:** Carlotta Isabella Zona, Martin H Fischer

**Affiliations:** Potsdam Embodied Cognition Group, Department of Psychology, University of Potsdam, Karl-Liebknecht-Strasse 24-25, House 14, OT Golm, 14476 Potsdam, Germany

**Keywords:** Spatial-numerical associations, Euclidean space, Manual pointing, Cognitive maps, Mental number line, Co-articulation, Human behaviour, Spatial memory

## Abstract

Number concepts are thought to be spatially organized along a mental number line increasing left-to-right (in Westerners) and bottom-to-top. However, the evidence for the emergence of horizontal and/or vertical spatial-numerical associations (SNAs) in manual pointing is mixed, and tasks implying magnitude-dependent number arrangements in physical space prevent conclusions about the emergence and extent of SNAs. Addressing these issues, we investigated SNAs with a two-step pointing task where the movement’s first step (provided after listening to a spoken number) always ended on the same central target displayed on a touchscreen. We analyzed spatial bias of this space-invariant, magnitude-independent first step. In the second step, participants localized the spoken number’s position in a clock-face arrangement, decoupling physical and numerical distance. In Experiment 1 (N = 20, spoken numbers from 1 to 12), pairwise numerical and Euclidean distances between (space-invariant) pointing locations were positively associated. Changes in magnitude of spoken numbers across successive trials yielded trends suggesting systematic pointing-location shifts. Experiment 2 (N = 20) presented 24 spoken targets (1–12.5, e.g., “three point five”) to increase clock-face salience. Both clock-face distance and numerical difference predicted Euclidean distances between pointing locations. In successive trials, positive magnitude changes resulted in leftward (clock-face congruent) and upward (MNL-congruent) shifts of pointing locations. The results suggest that numerical differences may be represented as 2D physical distances and support the emergence of vertical SNAs in manual pointing while avoiding previous experimental confounds. The findings align with the view that conceptual knowledge is represented in low-dimensional models that are spatially organized.

## Introduction

There has been a long tradition of associating conceptual knowledge with spatial representations. The classical method of loci was already taught in ancient Greece to boost memory performance by relating arbitrary content to a familiar spatial trajectory^1^. More recently, it has been proposed that spatialization of knowledge might be a neural necessity even for non-spatial facts, as learning and memory retrieval may critically engage hippocampal brain structures known to support spatial navigation^2–5^. Here we will examine behavioural predictions for numerical cognition derived from this important claim.

Numerical and mathematical relations have been understood in terms of two-dimensional space since before the introduction of Euclidean geometry^6^. Psychologically, numerical magnitudes are organized along a *Mental Number Line* (MNL)^7^, a one-dimensional continuum along which more similar magnitudes are represented closer to each other. This spatialization of number concepts along the MNL implies more cognitive overlap between direct number neighbors compared to more distant number pairs. This overlap explains why the former are harder to discriminate than the latter, a frequently replicated “distance effect” that obtains when comparing speed of judgments of more similar/dissimilar number magnitudes with lateralized buttons^8^. In other words, participants judge the larger/smaller of two numbers more quickly when choosing between 2 and 9 than when choosing between 2 and 4. Furthermore, when participants classify numbers as odd or even by pressing one of two lateralized buttons, Dehaene et al.^9^ observed facilitation of responses provided with the left button for smaller numbers, and conversely of right-button responses for larger numbers. This frequently replicated “spatial-numerical association” (SNA) has been attributed to small-left and large-right response mappings being congruent with the spatial organization of number concepts in representational space.

Similar evidence has been obtained for vertical SNAs (reviewed in^10^), traditionally using lateralized response buttons to compare speed of responses given with button mappings either congruent or incongruent with a vertical MNL, wherein smaller magnitudes are represented at the bottom and larger magnitudes at the top. For example, participants judge the larger/smaller of two numbers more quickly when smaller numbers are associated with lower-button responses and conversely larger magnitudes with upper-button responses. This association of larger numbers and upper space is interpreted as reflecting an experiential correlation of “up-is-more” that maps larger magnitudes onto higher locations. Together, this evidence has been taken to indicate that the MNL is itself oriented from left to right (especially in Westerners^11,12^) and from top to bottom.

However, results for the emergence of horizontal and/or vertical SNAs and their mutual relation are mixed, with some studies reporting a prevalence of horizontal over vertical associations^13–15^, and others reporting the opposite pattern^16–19^. Furthermore, it remains unclear why and how both horizontal and vertical associations should be entertained for the organization of the same representational concepts. A possible explanation may be found in work showing that the emergence of SNAs may be contingent upon specific task settings. For example, detecting SNAs may hinge on the required depth of numerical processing^15,20^, and importantly, on the degree of spatialization of the response space (e.g.,^21,22^). Indeed, using lateralized button presses typically limits responses either along the left–right or the down-up dimension when measuring SNAs. This may create methodological artifacts by generating spatial codes that would not emerge from space-invariant behavioral measures (for elaborations on this argument, see^23,24^), or by masking bi-dimensional associations.

Less constraining outcome measures were used very early in the history of SNA research. For example, Sir Francis Galton^25,26^ reported both horizontal and vertical number lines in free-hand drawings of adults who had been asked to visualize number sequences. More typically today, spatial and temporal aspects of manual pointing are studied to reveal cognitive processes related to knowledge representation and activation^27,28^. Pointing behaviour has been shown to replicate several established signatures of number processing, including the distance effect^29^ and SNAs^30^. Research examining numerical influences on spatial bias during manual pointing has often used number-to-position tasks, in which participants are asked to locate numbers on a visually presented number line (e.g., ref.^31–34^). Results have shown that participants tend to localize smaller numbers more to the left, and larger numbers more to the right than warranted, which is interpreted as support for the spatialization of number knowledge. However, this paradigm presents some shortcomings which may limit the conclusions to be drawn about number-knowledge representations. These issues are reviewed in what follows, alongside some research addressing them, before turning to the present study.

First, similar to lateralized response buttons, number-to-position tasks present the issue of limiting responses to one pre-established dimension (horizontal, vertical). This may introduce artificial mappings, and it prevents us from examining the relation of horizontal and vertical SNAs in 2D space.

Second, number-to-location tasks explicitly require participants to “show where” a number is, thus co-indexing number magnitude and space per task instructions. Importantly, explicitly implying such an overlap limits conclusions about the spatial nature of the representational format underlying numerical knowledge.

Third, in number-to-position tasks, target locations in the response space (i.e., the “correct” number locations) are perfectly confounded with numerical magnitude, and thus inter-item numerical differences directly correspond to physical distances between any two pointing targets in space (for an exception, see^31^). This prevents us from disentangling the effects of SNAs from the mappings imposed by the task. For example, the anticipation of upcoming motor targets is known as *co-articulation* and is a hallmark of goal-directed motor planning. Some examples include anticipatory finger movements during typing, pre-shaping of the phonatory system during speaking^28^, and postural adjustments at an identical intermediate target location depending on location of a subsequent target^35^. Of interest for us, number-to-position paradigms are often unable to dissociate the effects of co-articulation from the behavioral signatures of SNAs. For instance, finding leftward bias toward smaller and rightward bias toward larger magnitudes seems trivial if participants are explicitly instructed to point more leftward for smaller and rightward for larger magnitudes. More compelling would be MNL-congruency effects while participants are asked to always do the same (e.g., point to a space-invariant target).

A few studies addressing these methodological concerns used eye-tracking and spatial-localization paradigms to provide support for the emergence of SNAs in more conservative settings^13,36–40^. For example, Loetscher et al.^38^ and Viganò et al.^40^ monitored fixations to a blank screen during verbal random-number generation. Both reported that the direction and size of magnitude changes between successive numbers were preceded by fixation shifts mirroring the implied movement along both horizontal and vertical MNLs. Specifically, when participants generated a larger number in the current than the previous trial (e.g., a positive magnitude change from 5 to 10), their gaze shifted rightward and upward. Conversely, when the number produced in the current trial was smaller than in the previous trial (e.g., a negative magnitude change from 10 to 5), their gaze shifted leftward and downward.

Using a delayed spatial-localization task, Aulet et al.^13^ asked participants to reproduce the location of previously displayed Arabic numerals within a rectangular shape. Importantly, the task targeted memory for spatial location, making the outcome of interest fully independent of numerical magnitude. The results revealed an overall horizontal bias congruent with a left-to-right MNL, as smaller magnitudes were localized to the left of the target location, and conversely larger magnitudes were localized to the target’s right. In contrast, no evidence supporting vertical SNAs was found (see also^14^). However, the use of mouse-cursor data may be more suited to capture horizontal (over vertical) associations, as participants move the cursor only on the horizontal plane, while any motion along the sagittal axis is mapped onto the vertical dimension.

Summarizing, while support for horizontal (and some vertical) SNAs has been provided in settings addressing methodological issues, the evidence for horizontal/vertical SNAs and their mutual relationship remains mixed. What is more, the habitual practice of explicitly implying magnitude-dependent number arrangements in physical space—wherein physical and numerical distances have a 1:1 correspondence—prevents conclusions about the emergence and extent of SNAs in manual pointing measures.

The present study examines horizontal and vertical SNAs with a manual pointing task addressing these methodological concerns in two ways. Firstly, numerical magnitude was made irrelevant to the motor outcomes of interest by asking participants to listen to a spoken number and respond with a two-step pointing movement on a touch screen. The first step of the movement was always directed to the same centrally displayed location and was thus fully independent from the magnitude of the spoken number. The spatial coordinates of this first touch event constituted the outcome variable of interest. Secondly, in the second step of the movement, numerical and physical distances across pointing targets were partially decoupled by asking participants to localize the spoken number on a clock-face arrangement, rather than within a magnitude-based progression. For example, the numerical distance between 1 and 11 is 10 units, but their physical distance within the clock-face arrangement is 2 units. Dissociating MNL-congruent SNAs and the spatial mappings enforced by the clock-face response space enables us to disentangle the effects of co-articulation and numerical magnitude on manual pointing bias. Specifically, due to co-articulation, the endpoint of the movement’s first step is expected to be biased toward the endpoint of the movement’s second step, thus at least partially to reflect the clock-face arrangement.

Clock-face arrangements have already been used to document the flexibility of SNAs (although with the problematic provision of lateralized response keys), thus making it a promising medium for measuring the unbiased dimensionality of SNAs. First, Bächtold et al.^41^ showed left-to-right SNAs when their participants classified numbers as lengths (consistent with a previously presented ruler), while presenting a clock-face prior to the numbers induced right-to-left SNAs, consistent with their clock-face positions. More recently, Mingolo and colleagues^42^ showed that this reversal gradually increases with increasing salience of the clock-face.

To conclude, we asked whether the structural organization underlying numerical knowledge can be inferred from 2D pointing behavior while removing methodological confounds of manual pointing tasks. Within each trial, participants listened to a spoken number and pointed first to a centrally displayed circle on a touchscreen, then to the spoken number’s location in the clock-face arrangement. Note that the movement’s first target (the circle at the clock-face’s center) was displayed on-screen until a response was provided. The initial touch event was thus space-invariant, it imposed standardized motor-planning constraints to measure bias in a conservative setting, and it was independent of the spoken number. The spatial bias of this initial touch was our dependent variable. The current design improves on previous approaches, as it allows us to capture response patterns in two-dimensional space, and it renders numerical magnitude irrelevant to the pointing outcomes by studying a space-invariant response. Finally, the circular positioning of numbers on a clock-face is determined culturally, rather than representing a magnitude-based progression. Using a clock-face arrangement as response space thus allows us to dissociate the effects of co-articulation (i.e., the anticipation of the endpoint of the movement’s second step on its space-invariant first step) from SNAs induced by number-magnitude processing.

## Results

### Experiment 1

#### Numerical difference predicts Euclidean distance across pointing locations

Our first main hypothesis was that differences in numerical magnitude are cognitively represented as distances in Euclidean space, and that this may induce spatial biases in pointing toward a space-invariant location. Thus, pairwise numerical distance between spoken number targets was expected to be positively associated with the pairwise Euclidean distance between central pointing locations in response to each number. For instance, central pointing locations responding to number 12 should be more distant from locations responding to 1 than 11. This is because the numerical difference between 12 and 1 is greater than between 12 and 11, although both 1 and 11 are equally distant from 12 on the clock-face. To capture this, “clock-face distance” was added as a continuous predictor (range: 1–6, centered) reflecting the physical distance of the target numbers from one another in the clock-face space.

**Fig. 1:**
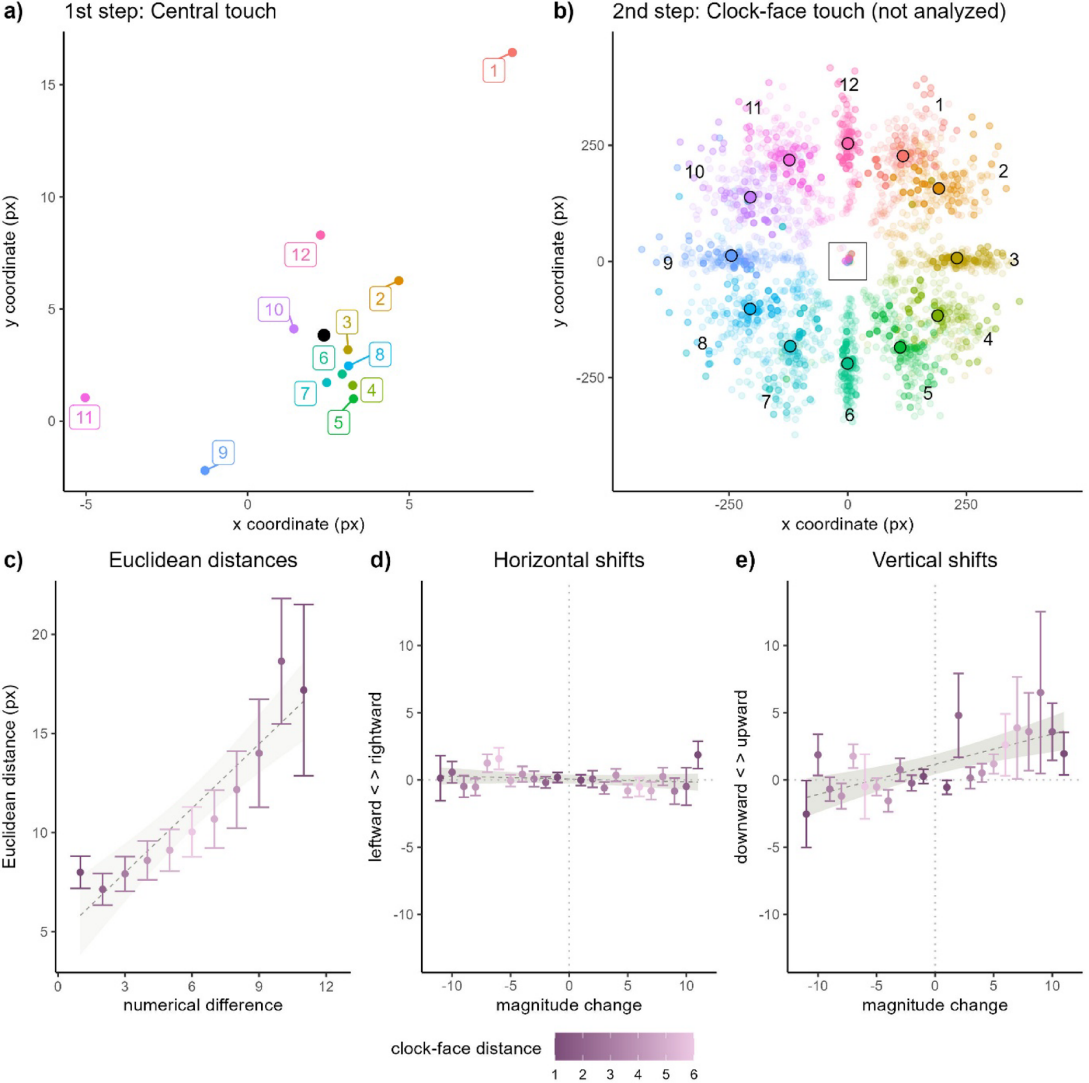
Results from Experiment 1. (**A**) Average end-locations of pointing toward the central dot. The black dot represents mean end-location across numbers. (**B**) End-locations of pointing to each number on the clock-face (not analyzed). Dots circled in black represent average end-locations by number. The central rectangle indicates the area represented in Panel A (not to scale). (**C)** Average pairwise Euclidean distance between central pointing locations associated with each target number as a function of pairwise numerical distance between target numbers. (**D**) Horizontal and (**E**) vertical shifts (in pixels) of central pointing location as a function of magnitude changes in successive trials (on the x-axis). Horizontal dotted lines correspond to no horizontal/vertical shifts from one trial to the next. Vertical dotted lines correspond to magnitude changes of zero. Darker colors correspond to shorter clock-face distances. Points correspond to mean values and error bars correspond to one SEM above/below the mean.

Following Aulet et al.^13^ and addressing the possibility of a linear association between numbers and space, we started by assessing whether smaller numbers resulted in leftward and/or downward bias of raw pointing coordinates, and larger numbers in rightward and/or upward bias (Fig. 1 Panel A). To do so, we analyzed x and y coordinates as a function of magnitude of the target number (continuous, centered) with mixed-effects linear regressions. Random effects enabled mean Euclidean distances and the effects of number magnitude to vary by participant. No linear relation of numerical magnitude and spatial bias of pointing coordinates was found (x position: *b* = − 0.07, *SE* = 0.06, *t* = − 1.29, *p* = 0.213; y position: *b* = 0.07, *SE* = 0.6, *t* = 1.12, *p* = 0.274).

Then, we tested our first main hypothesis by assessing the role of pairwise numerical and clock-face distances on pairwise Euclidean distances between pointing locations associated with each target number. This approach presents advantages over the analysis of raw coordinates toward each number because it enables us to capture two-dimensional spatial biases across movements requiring different physical endpoints. A linear mixed-effects regression was fitted to pairwise Euclidean distances across pointing locations in response to each number with pairwise numerical distance (continuous, centered) as main predictor of interest. Clock-face distance (continuous, centered) was included to account for the role of co-articulation, i.e., the anticipation of the endpoint of the movement’s second segment (Fig. 1 Panel B). Euclidean distances were log-transformed to satisfy the assumptions of our statistical approach.

The model revealed a positive association of numerical difference and Euclidean distance, indicating that the larger the numerical difference between spoken numbers, the larger the Euclidean distance between average pointing locations in response to each number (*b* = 0.07, *SE* = 0.02, *t* = 4.67, *p* < 0.001). In contrast, no effect of co-articulation was detected, as pairwise clock-face distance showed no influence on Euclidean distance across pointing locations (*b* < 0.01, *SE* = 0.02, *t* = − 0.24, *p* = 0.811). The interaction of both factors was not significant and did not improve model fit (*b* < 0.01, *SE* = 0.01, *t* = − 0.66, *p* = 0.509). A sensitivity analysis showed that the model achieved high statistical power for the effect of interest (1-β = 0.97).

These results support our first hypothesis and show that pairwise numerical differences across targets predicted Euclidean distance across pointing locations toward each number beyond the physical distance between any two targets in the clock-face arrangement.

#### Magnitude changes may induce numerical shifts in pointing locations

Our second hypothesis was for changes in magnitude across successive trials to be associated with directional shifts of pointing locations, following Loetscher et al.^38^ and Viganò et al.^40^. Magnitude changes were computed as the signed difference of the number in the current trial minus the number in the previous trial. For example, if the target number changed from 3 in the previous trial to 8 in the current trial, this was coded as + 5, or a positive magnitude change of 5 units. Conversely, if the number in the previous trial was larger than the in current trial (e.g., from 10 in the previous trial to 1 in the current trial), this corresponded to − 9, indexing a 9-unit negative change.

This analysis investigated the influence of magnitude changes in successive trials on shifts of x and y coordinates of pointing locations. In the horizontal dimension, a positive change in magnitude would typically be expected to yield rightward shifts of pointing locations. However, the clock-face context has been found to induce MNL-reversed SNAs, congruent with the clock-face mapping of larger numbers on the left and smaller numbers on the right^41,42^. We might thus expect to observe that more magnitude changes in successive trials produce MNL-incongruent (i.e., clock-face-congruent) horizontal shifts of pointing locations, with more positive (vs. negative) changes in target magnitude resulting in more leftward shifts in pointing locations. In the vertical dimension, more positive (vs. negative) magnitude changes were hypothesized to be associated with upward (vs. downward) shifts of pointing locations.

Signed differences in x and y coordinates of pointing locations were fitted to linear mixed-effects regressions with signed magnitude change across successive trials (continuous, centered) as main predictor. The random-effects structure included random intercept adjustments allowing the effects of magnitude change on coordinate shifts to vary randomly by number pair. Modeling participant-level variability resulted in convergence issues and was thus avoided.

In both horizontal and vertical dimensions, magnitude changes in successive trials were associated with non-significant numerical trends (Fig. 1, Panels D and E). In the horizontal dimension, there was some indication that more positive magnitude changes tended to be associated with leftward shifts (*b* = − 0.06, *SE* = 0.03, *t* = − 1.95, *p* = 0.066). Similarly, in the vertical dimension, more positive magnitude changes showed a trend toward an association with more upward bias (*b* = 0.19, *SE* = 0.10, *t* = 1.95, *p* = 0.069). Compatible with these non-significant effect sizes, a sensitivity analysis showed that the models’ ability to detect the effects of interest (1-β) was only 0.44 and 0.50 in horizontal and vertical dimensions respectively.

### Interim discussion

In Experiment 1, we hypothesized that numerical distance between spoken numbers may be associated with greater Euclidean distance between pointing movements towards a centrally displayed target, and that magnitude changes of targets across successive trials might be associated with MNL-congruent shifts in vertical coordinates and clock-face-congruent shifts in horizontal coordinates of pointing.

The results from Experiment 1 revealed a positive association of numerical distance between spoken numbers and Euclidean distance between pointing locations associated with each number, in support of our first hypothesis. However, we detected no significant effects of magnitude changes on shifts of x and y coordinates—although the observed numerical trends were at least compatible with our predictions.

A likely explanation for our failure to detect reliable SNAs may be found in the work by Bächtold et al.^41^ and Mingolo et al.^42^. Namely, the clock-face context might have engendered clock-face-congruent SNAs, in which the spatial-numerical mappings typical of the horizontal MNL are reversed. Similarly, the bottom-to-top associations of the vertical MNL are only maintained for the second half of the number range (i.e., 6 to 12). Hence, it is possible that the co-occurring and opposite effects of MNL-induced and clock-face-induced SNAs may have cancelled each other out in the current setting, resulting in no reliable patterns. This possibility was pursued in Experiment 2.

### Experiment 2

In Experiment 2, the salience of the clock-face environment was increased by asking participants to locate 24 instead of 12 number targets, ranging from 1 to 12.5 in 0.5-unit increments, which required both more fine-grained spatial processing of the response space, and the localization of number positions which are not automatically associated with clock-face locations (e.g., “three point five”).

#### Physical distance and numerical difference predict Euclidean distance

Regarding our first hypothesis, numerical differences across pairwise combinations of target numbers predicted Euclidean distances between average pointing locations toward each number in Experiment 1. Having increased the salience of the clock-face in Experiment 2, the physical distance across targets in the clock-face arrangement may be expected to predict Euclidean distance across average pointing locations toward each number (while it had no influence on Euclidean distances in Exp. 1).

**Fig. 2:**
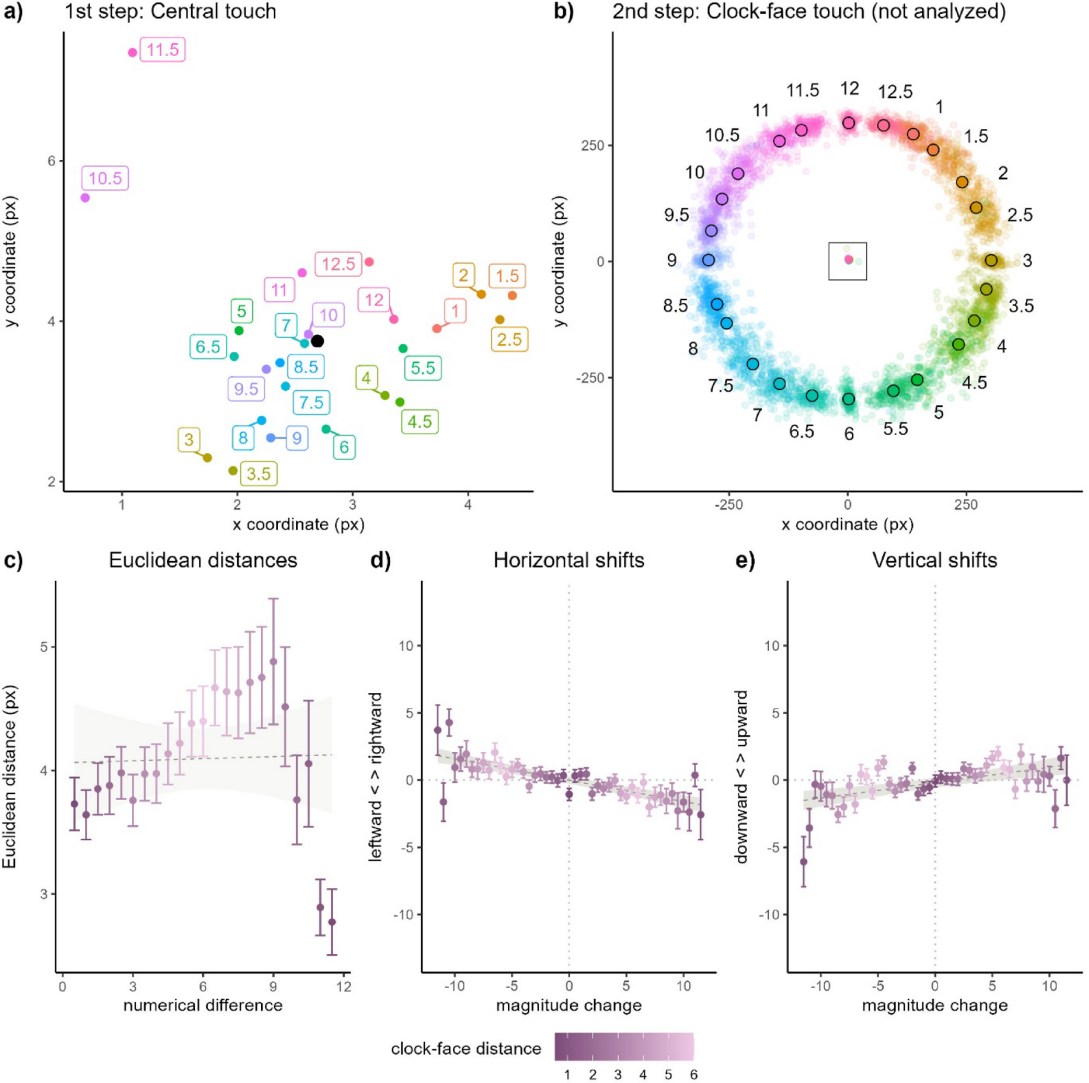
Results from Experiment 2. (**A**) Average pointing locations to the central dot for each target number. The black dot represents the average pointing location. (**B**) Pointing locations to the number’s position on the clock-face. Black-circled dots represent average pointing locations on the clock-face. The central rectangle indicates the area represented in A (not to scale). (**C**) Average pairwise Euclidean distances between pointing locations associated with each target number as a function of pairwise numerical distances between target numbers. (**D**) Horizontal and (**E**) vertical shifts (in pixels) of pointing location as a function of magnitude changes in successive trials (on the x-axis). Horizontal dotted lines correspond to no horizontal/vertical shifts from a trial to the next. Vertical dotted lines correspond to magnitude changes of zero. Darker colors correspond to shorter clock-face distances. Points correspond to mean values and error bars correspond to one SEM above/below the mean.

We again started by assessing the possibility of a linear association between number magnitude and spatial bias of raw pointing coordinates (Fig. 2, Panel A). The model’s structure was identical to Experiment 1. This time, increasing number magnitudes were associated with more leftward pointing coordinates in the horizontal dimension (*b* = − 0.15, *SE* = 0.04, *t* = − 3.85, *p* = 0.001) and more upward pointing coordinates in the vertical dimension (*b* = 0.07, *SE* = 0.03, *t* = 2.52, *p* = 0.021).

We then assessed the effects of numerical difference and clock-face distance on pairwise Euclidean distances between pointing locations associated with each number (Fig. 2, Panel C). The linear-regression model fitted to Euclidean distances of pointing locations across target pairs revealed small but significant effects of both numerical difference (*b* = 0.01, *SE* = 0.01, *t* = 2.43, *p* = 0.025) and clock-face distance (*b* = 0.02, *SE* = 0.01, *t* = 2.16, *p* = 0.045) on Euclidean distance.

A statistical comparison of the effect size of clock-face distance across both Experiments further confirmed that the manipulation increased clock-face salience in Experiment 2 compared to Experiment 1 (*b* = 0.05, *SE* = 0.02, *t* = 2.30, *p* = 0.027). The positive association of numerical difference and Euclidean distance was significant overall (that is, across Experiments: *b* = 0.04, *SE* = 0.01, *t* = 4.97, *p* < 0.001) and statistically reduced in Experiment 2 (vs. Exp. 1; *b* = − 0.06, *SE* = 0.02, *t* = − 3.53, *p* = 0.001).

#### Magnitude changes induce shifts in pointing locations

Turning the second hypothesis—on the role of positive/negative magnitude changes across successive trials on directional shifts of pointing locations—increasing clock-face salience was expected to result in significant clock-face-congruent (i.e., MNL-reversed) horizontal SNAs. This hypothesis was based on work by Mingolo et al.^42^, which showed progressive re-mapping of SNAs with increasing clock-face salience. In our setting, this should be indexed by more positive (vs. negative) magnitude changes yielding more leftward (vs. rightward) bias. In the vertical dimension, we expected to observe MNL-congruent, bottom-to-top associations (which were non-significant in Exp. 1).

These expectations were largely supported, with magnitude changes across successive trials predicting horizontal and vertical shifts of pointing locations (Fig. 2, Panels D and E). In the horizontal dimension, more positive magnitude changes were associated with more leftward bias and more negative changes conversely with more rightward bias (*b* = − 0.16, *SE* = 0.02, *t* = − 6.22, *p* < 0.001), reflecting the expected clock-face-induced, MNL-incongruent SNA. A sensitivity analysis showed that the model fitted to horizontal data reached high statistical power (1-β = 0.98).

In the vertical dimension, more positive magnitude changes were associated with upward shifts of pointing locations (*b* = 0.13, *SE* = 0.04, *t* = 3.06, *p* = 0.004), compatible with a bottom-to-top linear mapping of numerical magnitude. A sensitivity analysis showed that the model achieved appropriate power (1-β = 0.85). The observed effects were robust across both Experiments (n = 40; *b* = 0.18, *SE* = 0.07, *t* = 2.66, *p* = 0.010) and did not vary statistically across Experiments (*p* = 0.117).

## General discussion

The current study asked whether the structural organization underlying numerical knowledge can be inferred from manual pointing behavior while removing common methodological confounds of number-to-position tasks. Specifically, all responses followed the presentation of a spoken number and required first pointing to the center of the clock-face, then to the number’s location in the clock-face arrangement. The motor target of the movement’s first step (i.e., the center of the clock-face) was displayed on-screen until a response was provided, and the initial touch event was thus space-invariant and independent of the spoken number. We limited the analyses to the endpoints of the movement’s first step.

In Experiment 1, we found a strong positive association of numerical difference between pairwise combinations of number targets and Euclidean distance between pointing locations responding to each target. Changes in numerical magnitude across successive trials yielded non-significant numerical shifts of both horizontal and vertical pointing coordinates suggestive of right-to-left and bottom-to-top mappings.

In Experiment 2, the clock-face context was made more salient by increasing the targets numbers from 12 to 24 (e.g., 1, 1.5, 2). Euclidean distance between pointing locations in response to each target was predicted by clock-face distance–in contrast to Experiment 1. Analyses of horizontal and vertical shifts of pointing coordinates as a function of magnitude changes in successive trials showed significant clock-face-congruent (i.e., MNL-incongruent) horizontal associations in Experiment 2, alongside vertical SNAs indexing MNL-congruent bottom-to-top mappings. These effects were confirmed by compatible linear patterns of spatial bias in raw pointing coordinates, which were displaced more to the left and top regions of the response space with increasing numerical magnitude.

Next, we consider the theoretical implications of our results. First, the finding of a positive association of pairwise numerical difference and Euclidean distance between pointing locations in response to each number target (Exp. 1) provides novel evidence for an overlap in the cognitive representation of numerical difference and physical distance in two-dimensional space in both Experiments. While this association was smaller in Experiment 2, collapsing data from both Experiments showed a robust positive association of numerical and Euclidean distances overall (*p* < 0.001).

This result directly addresses the long-standing proposal that cognitive space may be isomorphic to physical space–i.e., that the functional relations among cognitive representations may parallel those among their external referents^43^. Within this cognitive representational space, similarity in relevant dimensions may be operationalized as spatial distance across items^44^. Critically, our results align with prior evidence for this claim while avoiding important methodological issues detailed above, including the explicit mapping of magnitudes onto pre-established spatial dimensions by task instruction. Our results thus provide support for the view that magnitude processing may implicate spatial processing^2–5^, resulting in SNAs even in tasks which require space-invariant responses, and dissociate magnitude from physical locations. Furthermore, the novel support for manual pointing signatures of SNAs in Euclidean space suggests that well-documented horizontal and vertical SNAs may reflect the same representation of number concepts in magnitude-based low-dimensional models.

Second, shifts of central pointing coordinates were modulated by the change in magnitude of spoken-number targets across successive trials. Our analytical approach followed prior research using blank-screen eye tracking^38,40^, and the results were largely consistent with previous reports of vertical and horizontal SNAs in patterns of uninstructed gaze behavior during random-number generation.

In the horizontal dimension, using a clock-face arrangement as response space resulted in a reversal of well-attested left-to-right mappings. This was only a non-significant trend in Experiment 1 and became significant as clock-face salience increased in Experiment 2. In other words, more positive magnitude changes were associated with leftward shifts of central pointing coordinates, and conversely more negative magnitude changes with rightward shifts, reflecting the horizontal right-to-left layout of numbers in the clock-face space. This evidence supports the role of the clock-face context on horizontal motor bias, replicating findings from Bächtold et al.^41^ and Mingolo et al.^42^ and extending their validity to manual pointing outcomes in two dimensions. Indeed, increasing the salience of the clock-face space in Experiment 2 resulted in a significant association of Euclidean distances and clock-face distance across pairwise combinations of numbers (which was not observed in Exp. 1). The association of Euclidean distance and numerical difference (which was highly significant in Exp. 1) was still significant in Experiment 2, though to a lesser extent. Crucially, we were able to measure strong horizontal SNAs in Experiment 2, which were congruent with the clock-face space and incongruent with the MNL. This suggests that the clock-face-congruent association may have been strong enough to override typically observed left-to-right associations in Experiment 2 but not in Experiment 1, consistent with prior evidence for the role of clock-face salience in determining size and direction of SNAs^42^.

In the vertical dimension, shifts of pointing coordinates were predicted by direction and size of magnitude changes in Experiment 2. More positive magnitude changes were associated with upward shifts and more negative magnitude changes with downward shifts, in line with previous eye-tracking evidence^38,40^.

The support for bottom-to-top SNAs in the vertical dimension provided with the current task settings is especially relevant for two reasons. For one, this finding contrasts with the results of Aulet et al.^13^, who reported no vertical associations of magnitude concepts from a pointing task addressing the same confounds as the current study. This discrepancy may be due to the use of mouse-cursor measures in^13^, in which participants only perform hand movements on a horizontal plane, and motion along the sagittal axis is translated into vertical cursor displacement. Our setting may thus have been better suited to study untransformed spatial behavior in the vertical dimension. Second, bottom-to-top and top-to-bottom mappings are both represented on the clock-face (on the left- and right-hand side, respectively)–in contrast to horizontal mappings, which are fully opposite to the left-to-right orientation of the MNL. Since both vertical mappings are represented in the response space, the observed preference for bottom-to-top mapping can be taken to index not task-induced spatial-spatial mappings, but rather the representational organization of number concepts in cognitive space, which may have rendered one (bottom-to-top) orientation of such concepts in vertical space predominant over the other (top-to-bottom).

Taken together, these results suggest that manual pointing reflected MNL-congruent mappings unless another (clock-face-congruent) SNA was made more salient, pointing to a degree of mutual exclusion, as well as flexibility, between conflicting space-number configurations.

In conclusion, the current findings corroborate the view, derived from methodologically problematic studies, that numerical differences may be cognitively represented as physical distances in Euclidean space, and that larger magnitude is associated with upper regions of space. Our results also confirm that the spatial organization of number knowledge may be inferred from manual pointing outcomes in task settings which do not explicitly co-index (pre-established) spatial dimensions and magnitude. In turn, the emergence of SNAs in space-invariant outcomes of manual pointing is compatible with the view that the organization of conceptual knowledge relies on mechanisms supporting navigation and memory for events in 2D space^2,5^, making spatialization a neurally plausible solution for the cognitive representation of non-spatial concepts such as numerical magnitude.

## Methods

### Experiment 1

#### Participants

The study was approved by the Ethics Committee of the University of Potsdam (Ethik-Antrag 5/2022) and conducted in accordance with the Declaration of Helsinki. 20 participants were recruited through the participant pool of the University of Potsdam and compensated with 8€/hour or university credit. Data from one participant had to be excluded due to a self-reported attention-deficit disorder. Data from one additional participant was thus collected and included to reach the planned sample size. Participants provided their informed consent to participate in the study.

The included 20 participants (11 females, 8 males, 1 non-binary; mean age = 24 years, SD age = 2 years) reported no history of motor or neurological conditions. Participants were mostly right-handed (on a scale from − 100—fully left-handed—to 100—fully right-handed: median = 91, mean = 68, SD = 53). All participants had first languages written left-to-right (16 German, 1 Russian, 1 Turkish, 1 Ukrainian), except for one speaker of Persian.

#### Apparatus, stimuli, and procedure

**Fig. 3:**
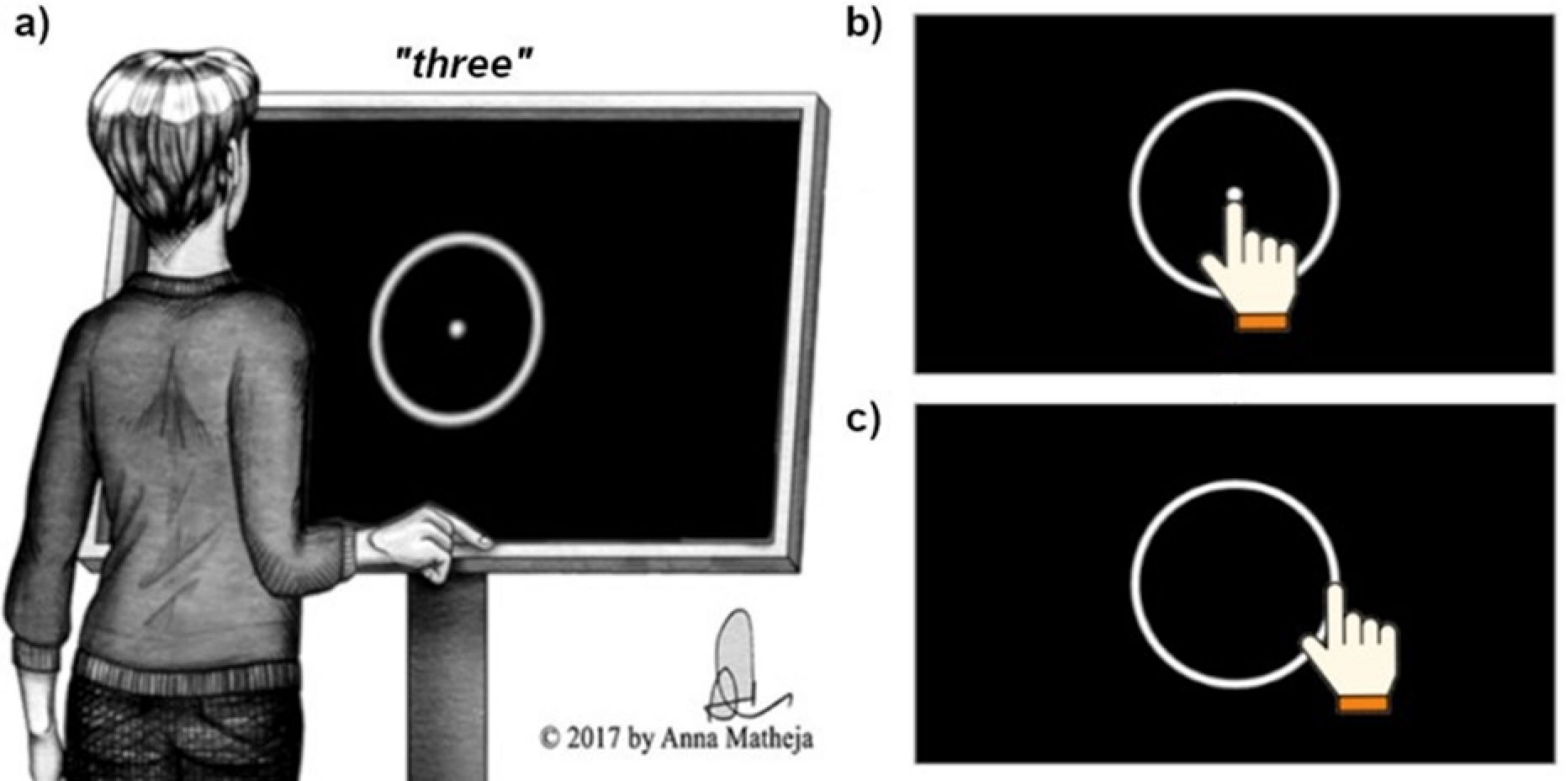
Experimental trial. (**A**) Participants stood in front of the touchscreen and listened to a spoken number between 1 and 12 (e.g., “three”). Figure adapted from^45^. (**B**) Participants pointed to the central dot. We analyzed responses to the central dot only. (**C**) Participants pointed to the number’s location as though on a clock-face.

Participants filled out a screening questionnaire with their demographic information, including their age, gender, handedness, and history of neurological, motor, or learning impairments. For the experimental task, participants stood at a comfortable distance from a 55-inch touchscreen (iiyama ProLite TH5563MIS, 1210 × 680 mm monitor size, 1920 × 1080pixels resolution, 60 Hz refresh rate) supported by a stand at a height of 1000 mm (lower edge). The screen was tilted by approximately 30° degrees for ease of use (Fig. 3, Panel A).

The screen displayed a central dot (radius = 20px) in white against a black background and a white ring around it (radius = 319px, width = 20px, Fig. 3). At the beginning of each trial, a spoken number (e.g., “three point five”) was presented using a single audio channel through a centrally located speaker. Spoken stimuli were created through a text-to-speech tool freely available online (www.ttsmp3.com) with a female voice speaking British English. The average stimulus duration was 455 ms (SD = 58 ms). The speaker’s volume was manually adjusted by the experimenter for the stimuli to be heard clearly by each participant individually.

Upon hearing the number, participants pointed first to the central dot, then to the number’s location on a clock-face arrangement. The analysis included data associated with the touch on the centrally displayed dot only (Fig. 3, Panel B). Data associated with the endpoint of the movement’s second step (Fig. 3, Panel C) was not further examined.

Each number was presented 10 times in fully randomized order. Note that the numbers on the clock-face were never presented visually. Before the task, participants completed one full round of pointing to all targets. Thus, participants saw a total of 132 trials. The task lasted about 10 min, and the duration of the experimental session did not exceed 20 min. All participants were instructed to respond with their right hand.

All statistical analyses were conducted in RStudio (version 4.2.3^46^). Linear regression models were fitted with *lme4* and *lmerTest* packages^47,48^. Visualizations were created with the *ggplot2* package^49^.

### Experiment 2

#### Participants

An independent sample of 20 participants was recruited through the participant pool of the University of Potsdam and compensated with 8€/hour or university credit to take part in Experiment 2.

Participants (13 females, 6 males, 1 non-binary; mean age = 25 years, SD age = 9 years) reported no history of motor or neurological conditions and were mostly right-handed (from − 100—fully left-handed—to 100—fully right-handed: median = 91, mean = 78, SD = 44). All participants’ first languages were written left-to-right (1 English, 13 German, 1 Greek, 1 Polish, 1 Russian, 1 Telugu, 1 Turkish) except for one first-language speaker of Arabic.

#### Apparatus, stimuli, and procedure

The experimental setup was identical to Experiment 1, except that the spoken numbers presented to participants ranged from 1 to 12.5 in 0.5-unit increments (e.g., “three point five”). The 12 additional spoken stimuli were added to the stimulus set from Experiment 1. The average duration of decimal number stimuli was 1117 ms (SD = 88 ms). Each number was presented 10 times and once during the practice trials, resulting in 264 trials. The experimental task lasted between 15 and 20 min, and the whole session did not exceed 30 min. All participants were instructed to respond with their right hand.

## Data Availability

Anonymized data, analysis pipeline, and figures are available on OSF [https://osf.io/me6tz] (https:/osf.io/me6tz).
